# Cardioprotective action of apocynin in isoproterenol‐induced cardiac damage is mediated through Nrf‐2/HO‐1 signaling pathway

**DOI:** 10.1002/fsn3.4465

**Published:** 2024-09-24

**Authors:** Md. Mizanur Rahman, Mirza Alimullah, Tahmina Yasmin, Nasrin Akhter, Iqbal Ahmed, Ferdous Khan, Mousumi Saha, Mohammad A. Halim, Nusrat Subhan, Md. Areeful Haque, Md. Ashraful Alam

**Affiliations:** ^1^ Department of Pharmaceutical Sciences North South University Dhaka Bangladesh; ^2^ School of Pharmacy and Public Health Independent University Dhaka Bangladesh; ^3^ Pharmacy Discipline Khulna University Khulna Bangladesh; ^4^ Division of Computer‐Aided Drug Design The Red‐Green Research Centre Dhaka Bangladesh; ^5^ Department of Chemistry and Biochemistry Kennesaw State University Kennesaw Georgia USA; ^6^ Department of Pharmacy International Islamic University Chittagong Chattogram Bangladesh

**Keywords:** apocynin, cardiac remodeling, inflammation, isoproterenol, oxidative stress

## Abstract

This investigation evaluated the therapeutic benefit of apocynin in isoproterenol (ISO)‐induced cardiac damage in rats. ISO‐administered male Wistar rats were treated with apocynin for 2 weeks. Blood plasma and left ventricle of heart tissues were collected and analyzed for oxidative stress‐related parameters such as malondialdehyde (MDA), advanced oxidation protein product (AOPP), and nitric oxide (NO). The activities of endogenous antioxidant enzymes such as superoxide dismutase (SOD) and catalase were also measured. The gene expressions of oxidative stress‐related proteins such as Nrf‐2, HO‐1, and HO‐2 in cardiac tissues were also measured. In silico studies like molecular docking and molecular dynamics were also performed to detect how apocynin interacts with NADPH and nitric oxide synthase at the molecular level. This investigation revealed significant elevation of serum transferase enzymes and creatinine kinase‐Muscle Brain (CK‐MB) activities in ISO‐administered rats compared to the control. Apocynin effectively normalized the serum transferases and CK‐MB activities in the blood of ISO‐stressed rats. Moreover, ISO‐induced elevations of MDA, NO, and AOPP levels were also suppressed by apocynin treatment. Consistently, apocynin restored the reduced SOD and catalase activities in ISO‐administered rats. This restoration of enzyme activity might be due to the increased expression of Nrf‐2 and HO‐1 and reduced expression of iNOS and TNF‐α in ISO‐administered rats. Histological analysis revealed that apocynin treatment ameliorated the mononuclear cell adherence and fibrosis in the cardiac tissue of ISO‐administered rats. Computational studies also support the experimental findings. This study demonstrates that apocynin prevents ISO‐induced cardiac injury not only by preventing inflammation but also by empowering the antioxidant defense system.

## Graphical abstract

This study suggests that apocynin protects ISO‐induced heart damage by preventing inflammation and fortifying the antioxidant defense system.

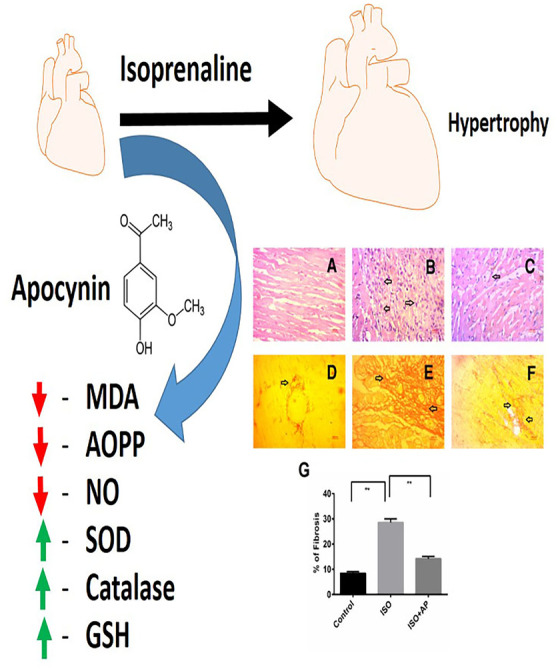

## 1 INTRODUCTION

Cardiovascular diseases were seen as the diseases of affluent societies; however, acute myocardial infarction (MI) is prevalent in many countries irrespective of economic status (Joseph et al., 2017). Ischemia and necrosis of the cardiac muscle, leading to MI, occur when the oxygen demand in the heart is increased dramatically (Heusch & Gersh, 2016; Prabhakaran et al., 2016). Tissue damage in the heart is also an outcome of inflammatory responses after an ischemic event (Heusch & Gersh, 2016; Prabhu & Frangogiannis, 2016). The release of pro‐inflammatory cytokines and the overproduction of reactive oxygen species (ROS) due to the infiltration of neutrophil may contribute to the damage of the cardiomyocytes (Prabhu & Frangogiannis, 2016). The beta‐adrenoceptor agonist isoproterenol (ISO), at a higher dose, triggers the overproduction of reactive oxygen species (ROS) like peroxide, superoxide, nitric oxide, and hydroxyl radicals in the myocardium (Afanas'ev, 2011). The increased amount of free radicals causes oxidative damage to lipid, protein, and DNA which eventually culminate in the irreversible injury of the cardiomyocytes and leads to the pathogenesis of myocardial infarction (Khalil et al., 2015; Ulla et al., 2017). ISO triggers oxidative stress in the myocardium of experimental animals; thus, it is frequently used to develop a drug‐induced animal model of MI (Sagor et al., 2015). Previous investigations reported that the efficacy of antioxidant enzymes is compromised in the infarcted zone of the heart (Karthikeyan et al., 2007; Khaper et al., 2003; Li et al., 2012; Münzel et al., 2015; Wei et al., 2017). Moreover, MI causes marked upregulation of inducible nitric oxide synthase (iNOS) which ultimately causes increased level of nitric oxide (NO) and peroxynitrite radicals (^·^ONOO^−^) (Afanas'ev, 2011; Yu et al., 2018). Accumulating proof also suggests that, in the infarcted zone, pro‐inflammatory cytokines like tumor necrosis factor‐alpha (TNF‐α) and interleukin‐6 (IL‐6) appear together with increased iNOS expression (Saini et al., 2005). Moreover, a key enzyme named nicotinamide adenine dinucleotide phosphate oxidase (NADPH oxidase) is overexpressed in the failing heart and acts as a significant catalyst for ROS production (Afanas'ev, 2011). On the other hand, earlier reports showed that during ischemic condition, nuclear factor (erythroid‐derived 2)‐like‐2 (Nrf‐2) expression‐mediated signaling provides defense to oxidative stress by enzyme induction which includes increased biosynthesis and activity of antioxidant enzymes (Shanmugam et al., 2019; Yu et al., 2015). In addition, Nrf‐2 regulates antioxidant genes by improving the function of heme oxygenase (HO‐1) during cardiac failure (Zhang et al., 2020). Moreover, significant cardioprotection has been observed through this pathway due to the improvement of the SOD and catalase activities during metabolic disorders (da Costa et al., 2019; Zhu et al., 2005).

Natural products and isolated compounds have shown considerable cardioprotection and benefits in oxidative stress‐related diseases (Abushouk et al., 2017; Kabir et al., 2022). Allicin showed cardioprotection in doxorubicin‐induced cardiotoxicity by improving the antioxidant enzymes and prevented the lipid peroxidation in the heart (Abdel‐Daim et al., 2017). Quercetine showed promising antioxidant and neuroprotective action discussed in a recent review revealing its mechanism of action in tissue protection as an important antioxidant (Grewal et al., 2021). Considering these literature, it is understanable that phytochemicals may contribute to the treatment process of cardiac disorder and other related oxidative stress‐related diseases (Abushouk et al., 2017). Apocynin is a small molecule that showed anti‐inflammatory and antioxidant activities (Simons et al., 1990; Stefanska & Pawliczak, 2008). It is also acting as an inhibitor of NADPH oxidase which may prevent superoxide generation (Sovari et al., 2008; Van den Worm et al., 2001; Ximenes et al., 2007). The beneficial effects of apocynin in the cardiovascular disorders are reported previously (Gimenes et al., 2018; Liu et al., 2015; Saleem et al., 2018; Sovari et al., 2008). An earlier investigation suggests that apocynin prevents oxidative stress‐mediated lipid peroxidation with the restoration of the endogenous antioxidant enzymes in the myocardium of ISO‐administered rats (Tanriverdi et al., 2017). The administration of apocynin also prevented the rise of NADPH oxidase expression and hypertrophic signal‐related genes in heart of ISO‐administered rats (Saleem et al., 2018). Apocynin also prevented multiple nephrectomy‐induced elevations of malondialdehyde and increased activity of NADPH oxidase and thus ameliorated fibrosis in the cardiac tissue (Liu et al., 2015). Apart from the above information, apocynin also possesses beneficial effects in the management of diabetes (Gimenes et al., 2018), asthma, lung injury (Al‐Mehdi et al., 1998; Pearse & Dodd‐o, 1999), and stroke (Tang et al., 2007). However, limited information is found regarding the role of apocynin in preventing ISO‐mediated detrimental activation of iNOS and associated oxidative damage in the heart. By considering the defensive role of apocynin in the above‐mentioned literature, this study was considered to delve deeper into the cardioprotective effects and its mechanism of action against ISO‐administered cardiac damage in rats.

## 2 MATERIALS AND METHODS

### 2.1 Chemical and reagents

Standard malondialdehyde, 1,1,3,3‐tetra methoxy propane (PubChem CID 66019), thiobarbituric acid (TBA) (PubChem CID 2723628), hydrogen peroxide (PubChem CID 784), chloramine‐T (PubChem CID 3641960), sodium nitrate (PubChem CID 24268), and trichloroacetic acid (PubChem CID 6421) were purchased from Merck, Germany. Isoproterenol hydrochloride (PubChem CID 5807) was obtained from Loba Chemie, India. RNA purification kit, cDNA synthesis kit, SYBR premix, and primers were obtained from Thermo‐Fisher Scientific (Massachusetts, USA). Other reagent‐grade chemicals were purchased from authentic and validated sources in India and China.

### 2.2 In silico molecular interaction study of apocynin with NADPH oxidase and nitric oxide synthase

After obtaining the molecular structure of apocynin from the Drug Bank, the structure was converted into PDB format for further processing. The vibrational frequency was calculated by density functional theory (DFT) using Gaussian 09 program package after structure optimization (Frisch et al., 2009; Lee et al., 1988). For all calculations, 6–31 + G(d, p) basis set was used. The ligand was also prepared by adding Gasteiger charge and hydrogen atoms. The three‐dimensional structures of iNOS and NADPH oxidase were collected from Protein Data Bank (PDB) database (PDB ID: 1DD7; Chain A) (McMillan et al., 2000). Before docking, removal of heteroatoms and water molecules and addition of hydrogen atoms and Kollman charges were done using PyMol (version 1.3) software package. For docking analysis, AutodockVina was used (Morris et al., 1998). The prepared proteins and ligands were then uploaded to the CB‐Dock2 server for structure‐based blind docking analysis using default parameters. Docking scores as kcal/mol, cavity volume, grid area, and docking residues were obtained from the analysis. Then, the 2D images of the interaction profiling were generated by BIOVIA Discovery Studio (Version 21). The binding affinities of apocynin to Nfr2 and NADPH oxidase were expressed as kcal/mol.

The docked ligand–receptor system was also employed for molecular dynamics (MD) simulation using the AMBER14 force field within the YASARA Dynamics program (Krieger & Vriend, 2015). The simulation was carried out under NPT conditions, impersonating physiological settings with a temperature of 298 Kelvin, a pH of 7.4, and a 0.9% NaCl concentration. Steepest gradient approach followed by simulated annealing method was used to minimize initial energy for each simulation performed. For the overall simulations, a time step of 1.25 fs was used. Finally, 45 ns MD simulation was carried out for each system. For analysis purpose, every 100 ps were saved.

### 2.3 Animals

For in vivo experiments, male adult (10–11 weeks old) Long Evans rats were obtained from the Central Animal House of North South University, Bangladesh. The rats were reared separately in standard‐size cages placed in an air‐conditioned room (temperature 25 ± 1°C, humidity 58%) with an accurate maintenance of 12 h/12 h light–dark condition with random access to food and water. The Internal Animal Care and Use Committee (IACUC) of the university reviewed and approved (IACUC No. 2019/OR‐NSU/IACUC No.0403) the methods of experiments and procedures of sacrifice.

### 2.4 Induction of cardiac damage through isoproterenol (ISO) injection

ISO was administered as subcutaneous injections (50 mg/kg) to induce cardiac damage (Sagor et al., 2015; Ulla et al., 2017). A total of 18 rats were divided into three experimental groups, each of which had six rats (*n* = 6).

Group I: Control rats consumed standard food and water.

Group II: 50 mg/kg SC injection of ISO twice a week.

Group III: 50 mg/kg SC injection of ISO twice a week together with oral administration of apocynin (100 mg/kg/day).

The ISO and apocynin administration was continued for 14 days, after which rats were euthanized by administering pentobarbital anesthesia (65 mg/kg *i.p*). Blood samples were taken in microcentrifuge tubes (1.5 mL), and plasma was separated by centrifugation (800 *g*). Then, the plasma was separated and stored at −20°C for further biochemical analysis. The cardiac muscle was also collected immediately, and part of it was placed in neutral buffered formalin (NBF) (pH 7.4) for histopathological analysis. Another part of the cardiac tissue was stored in a refrigerator at −20°C for further biochemical analysis.

### 2.5 Assessments of heart‐specific enzymes

The activities of cardiac function‐related vital enzymes such as CK‐MB, ALT, AST, and ALP were analyzed in plasma samples by using commercial kits obtained from DCI Diagnostics (Budapest, Hungary), according to the protocol described by the manufacturer.

### 2.6 Measurement of oxidative stress parameters

100 mg cardiac tissue sample from each heart was homogenized in 900 μL phosphate buffer (pH 7.4) and centrifuged at 3200 *g* for 30 min at 4°C. The resulting clear supernatant was collected in microcentrifuge tubes and was used for the analysis of oxidative stress‐related parameters like malondialdehyde (MDA), nitric oxide (NO), and advanced oxidation protein product (AOPP) according to the methods described in previous literature (Sagor et al., 2015; Ulla et al., 2017). The concentrations of MDA, NO, and AOPP were calculated from their corresponding standard curves. The concentrations of MDA and NO were expressed as nmol/mL, and APOP concentration was expressed as nmol/mL chloramine‐T equivalents.

### 2.7 Assessment of antioxidant enzyme activity and reduced glutathione (GSH) level

Superoxide dismutase (SOD) and catalase activities were also evaluated in the heart and plasma using methods mentioned earlier in the literature (Sagor et al., 2015; Ulla et al., 2017). Briefly, SOD activities were calculated by 50% inhibition of the auto‐oxidation of catecholamine. Moreover, an absorbance change of 0.01 in each minute was regarded as a unit change of catalase activity. The level of reduced glutathione (GSH) in the plasma and homogenized heart tissue sample was quantified by Ellman's reagent which involves the measurement of absorbance of a yellow‐colored anion of 2‐nitro‐5‐thiobenzoic acid (TNB) at 410 nm.

### 2.8 Evaluation of gene expression

GeneJET RNA Purification Kit of Thermo‐Fisher Scientific (Massachusetts, USA) was used for the total mRNA isolation and purification from the heart muscle. After checking the purity and measuring the concentration of mRNA, by NanoDrop (Thermo‐Fisher Scientific, USA), 1 μg mRNA from each sample was taken for the synthesis of cDNA using RevertAid First Strand cDNA Synthesis Kit of Thermo‐Fisher Scientific in a T100™ Thermal Cycler (Bio‐Rad, California, USA). This cDNA was then utilized to conduct quantitative real‐time PCR utilizing SYBR Green qPCR Master Mix of Thermo Scientific (USA). To quantify the transcript level of the target proteins, forward and reverse primers were designed by Primer3 program (Table 1). The real‐time PCR was performed according to a program developed by Khan et al. 2016, in a PCR system of Bio‐Rad Laboratories Inc. (California, USA). The data acquisition and analysis were done by CFX Manager™ developed by the same manufacturer. The mRNA level of each target gene was assessed by normalizing to the mRNA level of β‐actin, which served as a control for the same rat.

**TABLE 1:** The forward and reverse sequence of the primer used in this study.

Name of gene (GeneBank accession No.)	Type	Sequence
iNOS (NC_005102.4)	Forward	5′‐TGGTCCAACCTGCAGGTCTTC‐3′
	Reverse	5′‐CAGTAATGGCCGACCTGATGTTG‐3′
Catalase (NM_012520.2)	Forward	5′‐ATTGCCGTCCGATTCTCC‐3′
	Reverse	5′‐CCAGTTACCATCTTCAGTGTAG‐3′
MnSOD (NM_017051.2)	Forward	5′‐TCACCGAGGAGAAGTACCAC‐3′
	Reverse	5′‐TCCAGCAACTCTCCTTTGGG‐3′
Glutathione reductase (U73174.1)	Forward	5′‐GGGCAAAGAAGATTCCAGGTT‐3′
	Reverse	5′‐GGACGGCTTCATCTTCAGTGA‐3′
Heme oxygenase‐1 (HO‐1) (NM 012580.2)	Forward	5′‐CAGCTCTATCGTGCTCGCATG‐3′
	Reverse	5′‐TCCTCTGTCAGCAGTGCCT‐3′
Heme oxygenase‐2 (HO‐2) (NM_001277073.1)	Forward	5′‐GACCAAGGAAGCACATGACC‐3′
	Reverse	5′‐TGCTTCCTTCCGGTGTAGTT‐3′
Nrf‐2 (AF304364.1)	Forward	5′‐AGCGTGGAGAGATATGAGCC‐3′
	Reverse	5′‐ATCATCCGCCACTCATTCCT‐3′
IL‐6 (M26744.1)	Forward	5′‐AGCGATGATGCACTGTCAGA‐3′
	Reverse	5′‐GGTTTGCCGAGTAGACCTCA‐3′
TNF‐α (NM_012675.3)	Forward	5′‐ATGTGGAACTGGCAGAGGAG‐3′
	Reverse	5′‐CCACGAGCAGGAATGAGAAGAG‐3′
Collagen‐1 (BC133728.1)	Forward	5′‐CTGGTACATCAGCCCAAACCC‐3′
	Reverse	5′‐CGCAGGAAGGTCAGCTGGATAG‐3′
TGF‐β1 (AY550025.1)	Forward	5′‐AAGAAGTCACCCGCGTGCTA‐3′
	Reverse	5′‐TGTGTGATGTCTTTGGTTTTGTC‐3′
β‐Actin (NM_031144.3)	Forward	5′‐CCTCTATGCCAACACAGTGC‐3′
	Reverse	5′‐CCTGCTTGCTGATCCACATC‐3′

### 2.9 Histopathological studies

The hearts stored in neutral buffer formalin (NBF) were used for histological analysis. These preserved heart tissues have undergone a series of ethanol and xylene treatments before making the wax block. The processed heart tissues were made blocks by embedding them in paraffin wax and cut at five 𝜇m thickness using a microtome. Those slices of heart were stained by Hematoxylin and Eosin (H&E), and Sirius red (SR), using routine laboratory procedures. All stained slides were checked and photographed using an optical microscope (Zeiss Axio Scope) at 40× magnification. The Sirius red‐stained tissues were evaluated for fibrosis region using ImageJ software following a previously described method (Hasan et al., 2020).

### 2.10 Statistical analysis

Mean ± SEM was used to present the generated data in this study. One‐way analysis of variance (ANOVA) was performed for the statistical analysis, and posthoc test was chosen as Tukey. Statistical significance was considered as *p* < .05 in all cases, and differences are marked as asterisk marks. The single asterisk mark represents *p* < .05 level of significance and double asterisk marks represent *p* < .001 level of significance.

## 3 RESULTS

### 3.1 Binding behavior of apocynin with NADPH oxidase and nitric oxide synthase

**FIGURE 1:**
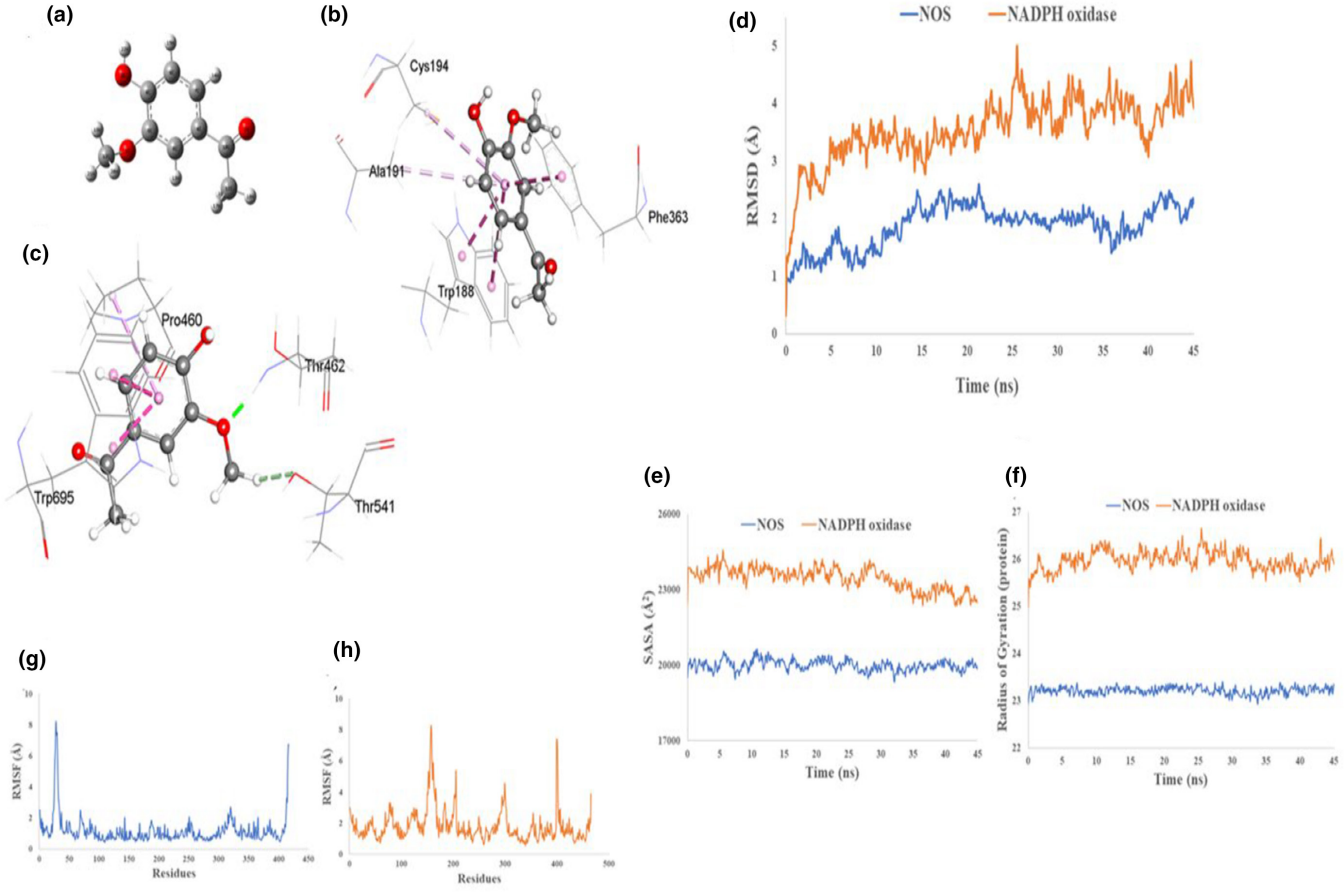
(a) Optimized structure of Apocynin in gas phase at B3LYP/6‐31+ G (d,p) level in Gaussian 09. Predicted non‐covalent interactions from docking of Apocynin with (b) NOS and (c) NADPH oxidase showing hydrogen bond interaction (green color), including π–π stacking (pink color). (d) The time series of the RMSDs of Cα atoms for proteins. Structural changes of protein by means of (e) solvent accessible surface area and (f) radius of gyration. The structural changes of protein, (g) NOS and (h) NADPH oxidase, by means of root means square fluctuation (RMSF) analysis.

In order to check the binding affinity of apocynin against the two proteins, molecular docking simulations were performed. The docking analysis showed that the binding affinities of apocynin with NADPH oxidase and NOS are −6.9 and −7 kcal/mol, respectively. The binding affinity and interaction obtained from molecular docking are shown in Table 2. NOS and NADPH oxidase both interact with apocynin by pi–pi stacking and pi–alkyl interactions (Figure 1). The number of hydrophobic interactions is more significant in the case of the NOS–apocynin complex. In contrast, two types of H‐bond, one with THR462 residue and other with THR541 residue, are observed only in NADPH oxidase–apocynin complex.

**TABLE 2:** Binding affinity (kcal/mol) and non‐covalent interactions of apocynin.

Receptor	Binding affinity (kcal/mol)	Hydrophobic			
		Bonding type	Protein	Ligand	Distance (Å)
			Interacting amino acids	Interacting atoms or rings	
NOS	−7.0	Pi–Pi Stacked	PHE363	Phenol ring	4.23003
			TRP188	Phenol ring	5.11264
			TRP188	Phenol ring	3.88102
		Pi–Alkyl	ALA191	Phenol ring	5.39679
			CYS194	Phenol ring	4.89971
NADPH oxidase	−6.9	Pi–Pi Stacked	TRP9695	Phenol ring	3.89168
			TRP695	Phenol ring	3.88562
		Pi–Alkyl	PRO460	Phenol ring	5.02654
		**Hydrogen Bonding**			
		Conventional	THR462	O…H–N	1.91109
		Carbon Hydrogen Bond	THR541	C–H…O	2.69934

For the understanding of binding affinity, structural configuration, and flexibility, MD simulations were performed on the docked complex. The atomic RMSDs of Cα atoms of proteins for both protein–ligand complexes were calculated and plotted as a function of time (Figure 1d). Figure 1d demonstrates the behavior of the proteins during the simulation. High fluctuation in RMSDs of Cα was found for NADPH oxidase where NOS showed lower RMSDs, suggesting higher stability in NOS–ligand complex in comparison with the NADPH oxidase, which was in agreement with docking results.

Root mean square fluctuation (RMSF), the radius of gyration, and solvent accessible surface area (SASA) of the protein were used to examine the configurational changes of two complexes (Figure 1e,f). Figure 1e represents the SASA of proteins, in which iNOS showed slight fluctuation in SASA but NADPH oxidase showed decreased SASA, indicating less compact protein structure. Additionally, the values of the radius of gyration analysis (Figure 1f) revealed that NADPH oxidase possessed a larger radius of gyration, which also indicate less compact protein structure. Thus values of SASA examination and radius of gyration studies validate the findings of each other.

Figure 1g,h represents the calculated RMSF values for both protein–ligand complexes from the trajectories. RMSF reflects the flexibility of each deposit in the proteins. Higher fluctuations were observed in the case of NADPH oxidase compared to NOS, ranging from 147 to 168, 200 to 210, 288 to 304, 398 to 404, and 456 to 465 in case of NADP oxidase.

### 3.2 Effect of apocynin on heart‐specific enzyme activities in ISO‐induced rats

**FIGURE 2:**
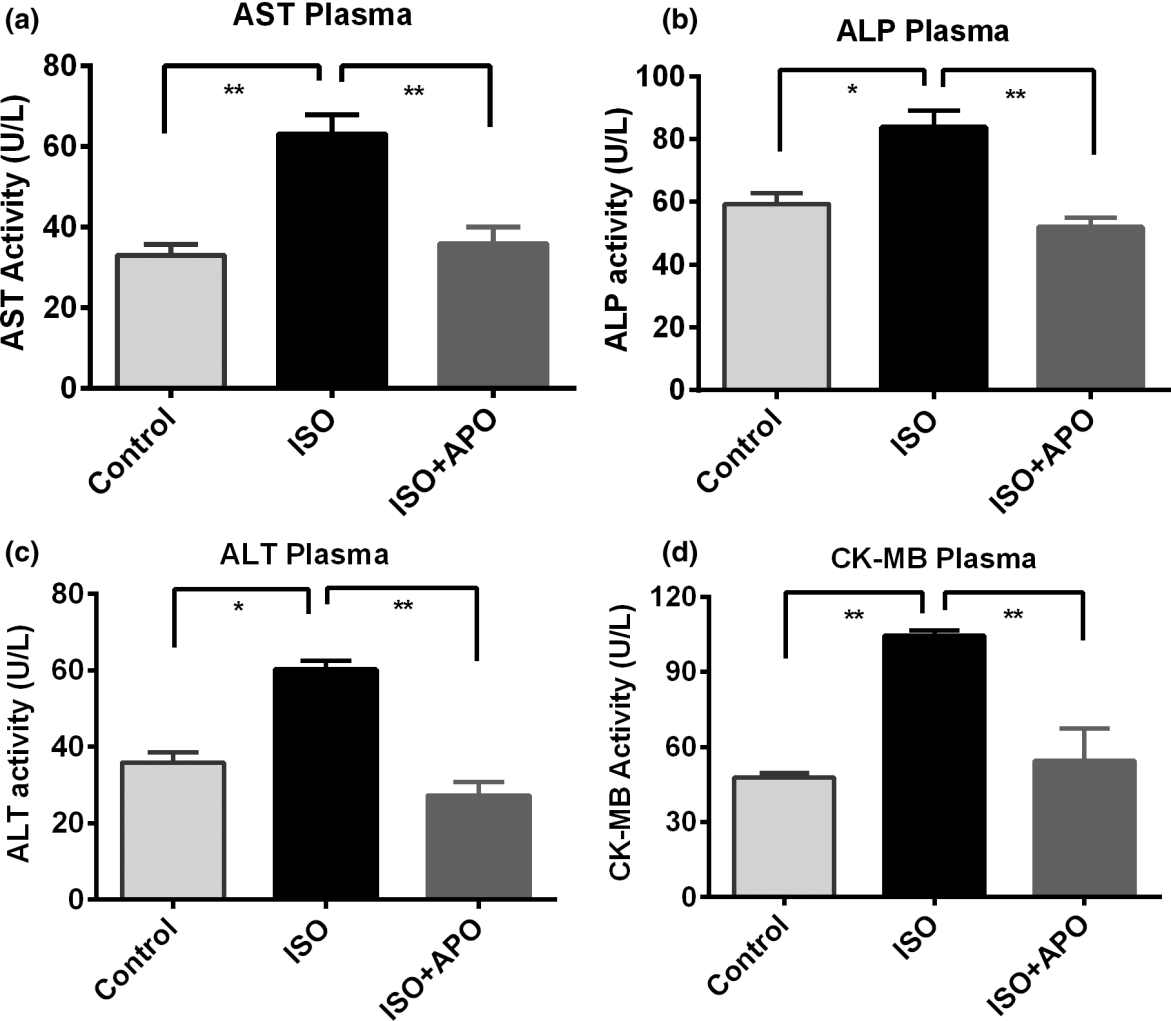
Effect of apocynin (APO) *on* AST, ALP, ALT and CK‐MB activity in plasma of ISO‐administered rats. Data are expressed as mean ± SEM, *n* = 6. Statistical analysis was done by one‐way ANOVA followed by Tukey's post hoc test. Statistical significance was considered as *p* < .05 and marked as asterisk mark. In case of *, *p* < .05 and **, *p* < .001.

The transaminase (AST) activity was found significantly (*p* < .001) higher in ISO‐administered rats in comparison to the control rats. Apocynin treatment significantly reduced the activity of AST which was elevated due to ISO administration (Figure 2a). ISO administration also amplified the ALP and ALT activities markedly (*p* < .05) in plasma which were also reduced significantly (*p* < .001) due to the apocynin treatment (Figure 2). Moreover, ISO administration in rats increased cardiac tissue‐specific CK‐MB activity significantly (*p* > .001) in plasma compared to the control rats (Figure 2). Apocynin treatment also significantly (*p* < .001) prevented the ISO‐induced increased CK‐MB activity (Figure 2).

### 3.3 Effect of apocynin on oxidative stress

**FIGURE 3:**
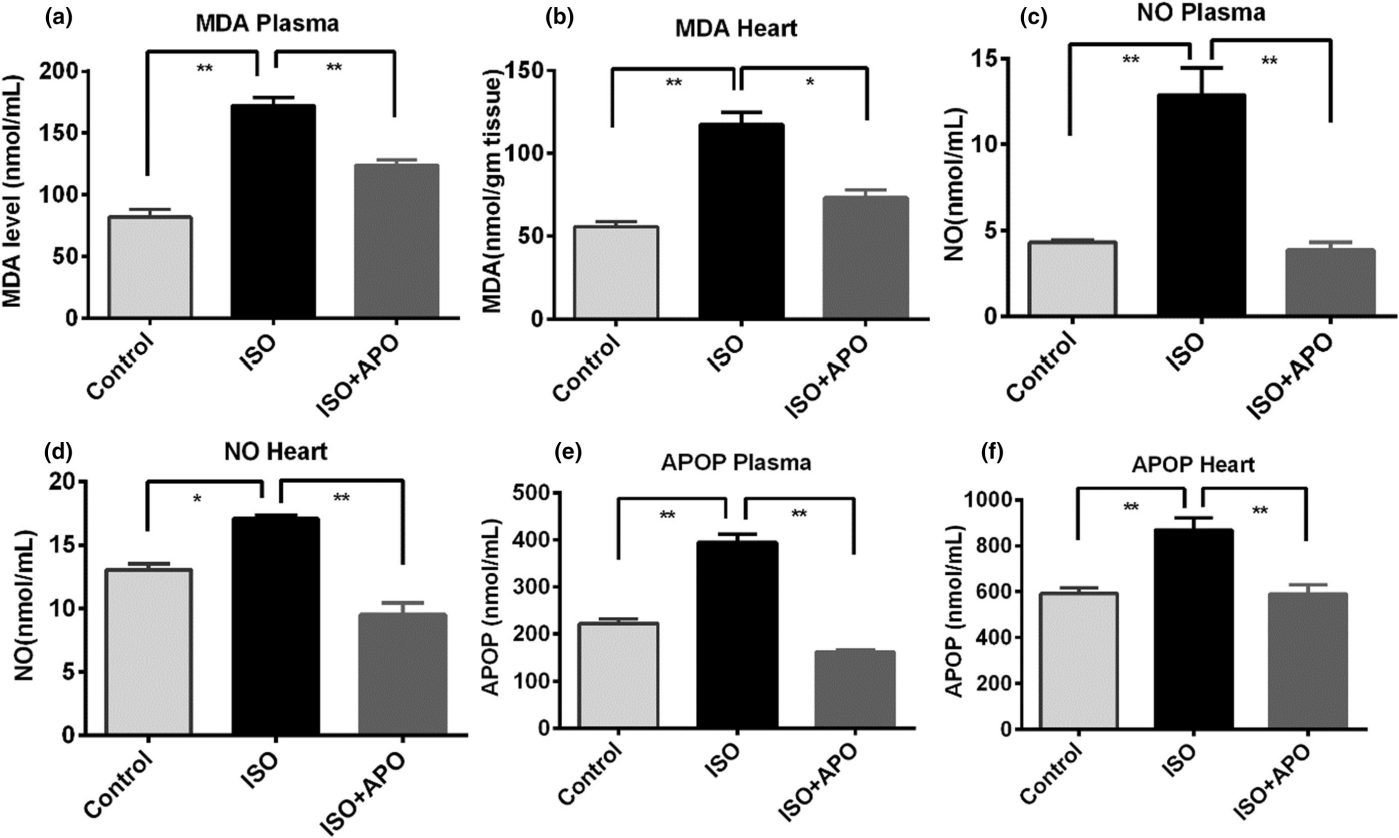
Effect of Apocynin (APO) on oxidative stress parameters MDA, NO, and APOP in plasma and heart tissue homogenates of ISO‐administered rats. Data are expressed as mean ± SEM, *n* = 6. Statistical analysis was done by one‐way ANOVA followed by Tukey's post hoc test. Statistical significance was considered as *p* < .05 and marked as asterisk mark. In case of *, *p* < .05 and **, *p* < .001.

For evaluating the effect of apocynin on oxidative stress in ISO‐administered rats, we analyzed MDA, NO, and AOPP levels in plasma and heart homogenates. This part of our study showed that subcutaneous administration of ISO significantly increased the lipid peroxidation as measured by the concentration of MDA significantly (*p* < .05), both in the plasma and in the cardiac tissues of the rats. Lipid peroxidation was prevented by apocynin treatment as shown by the decreased level of MDA in the heart and plasma of ISO‐administered rats (Figure 3). ISO administration also increased the NO level in the plasma and heart significantly (*p* < .05) compared to the control rats (Figure 3). Apocynin treatment normalized the NO level in the plasma of a heart of ISO‐administered rats (Figure 3). Moreover, ISO administration in rats showed a significant (*p* < .05) rise in AOPP level in the plasma and heart compared to the control rats (Figure 3). Apocynin treatment also decreased the elevated level of AOPP in the heart and plasma of ISO‐administered rats (Figure 3).

### 3.4 Effect of apocynin on antioxidant activities

**FIGURE 4:**
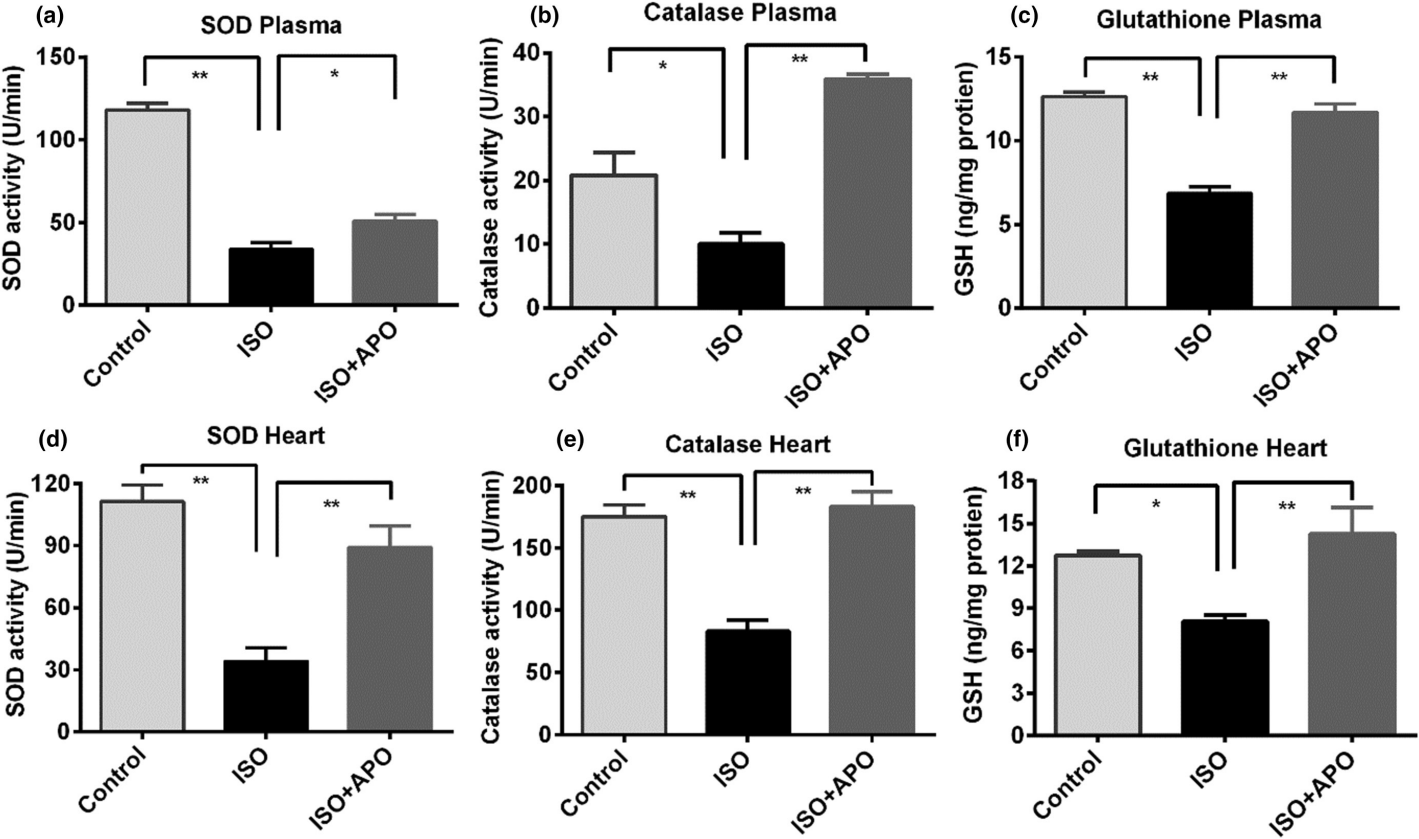
Effect of apocynin (APO) *on* SOD activity, catalase activity, and reduced glutathione level in plasma and heart of ISO‐administered rats. Data are expressed as mean ± SEM, *n* = 6. Statistical analysis was done by one‐way ANOVA followed by Tukey's post hoc test. Statistical significance was considered as *p* < .05 and marked as asterisk mark. In case of *, *p* < .05 and **, *p* < .001.

In this section of our study, both plasma and heart tissues were used to assay the catalase and superoxide dismutase (SOD) activities. ISO administration enfeebled the activity of SOD and catalase not only in the plasma but also in the heart significantly (*p* < .001) (Figure 4a,d). Treatment with apocynin caused significant increase in the activity of both SOD and catalase in both the plasma and heart. Similarly, ISO administration caused significant depletion in the level of reduced glutathione both in plasma (p < .05) and in heart (*p* < .001). We found that the feeding of apocynin completely restored the level of reduced glutathione (Figure 4c,f).

### 3.5 Gene expression of antioxidant enzymes

**FIGURE 5:**
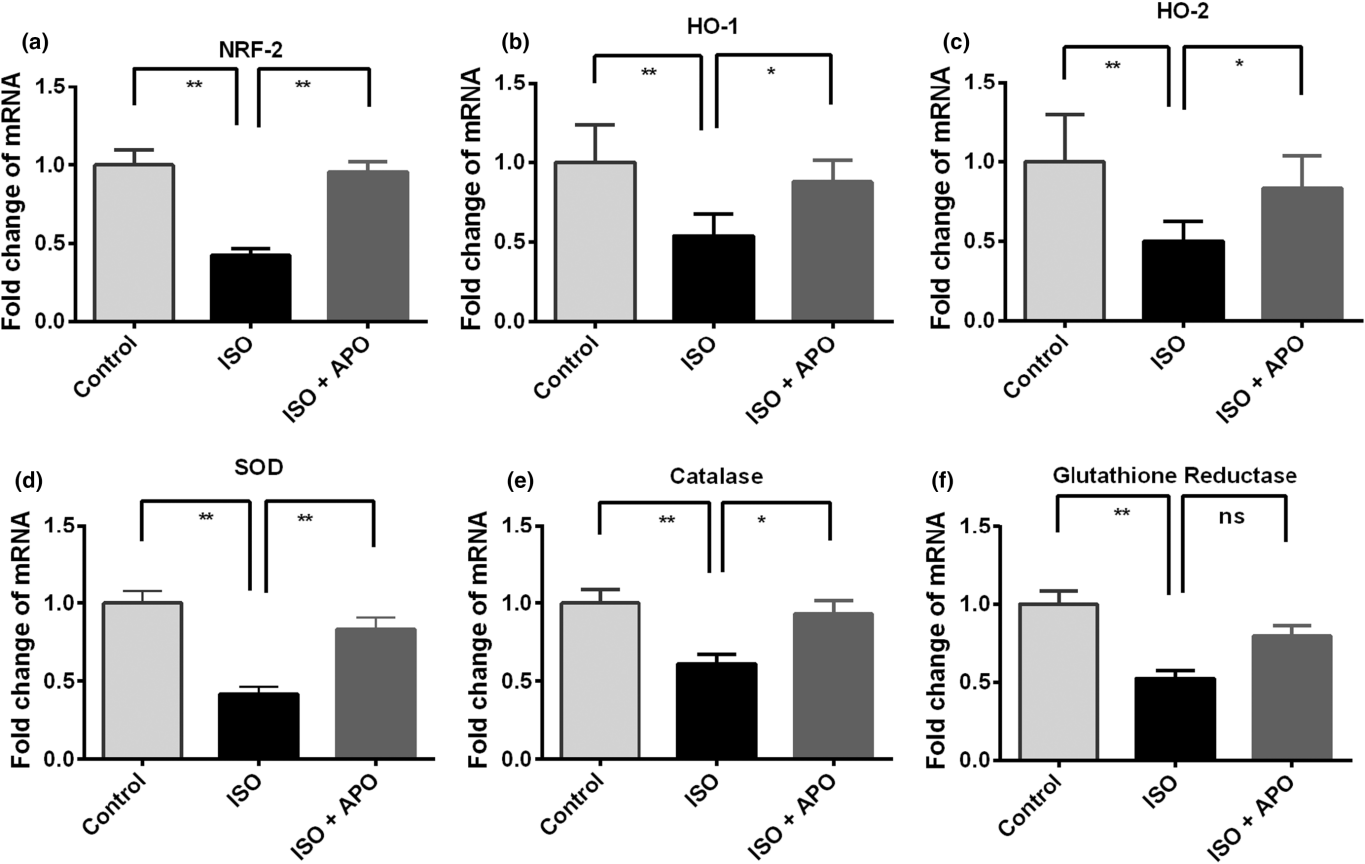
Effect of apocynin (APO) on gene expression related to antioxidant enzymes in the heart of ISO‐administered rats. Data are expressed as mean ± SEM, *n* = 6. Statistical analysis was done by one‐way ANOVA followed by Tukey post hoc test. Statistical significance was considered as *p* < .05 and marked as asterisk mark. In case of *, *p* < .05 and **, *p* < .001.

The antioxidant and anti‐inflammatory enzymes gene expression were regulated by the Nrf‐2 which is a multifunctional cytoprotective protein and thus acts as a potent endogenous defense against oxidative stress‐induced inflammation. Therefore, in this part of our study, we considered exploring the effect of apocynin on ISO‐mediated change of Nrf‐2 expression. Here, we noticed significantly (*p* < .001) lower transcript level of Nrf‐2 in the ISO‐administered group in comparison to the control rats (Figure 5a). Subsequently, in the downstream, the gene expression of both inducible and constitutive isoforms of hemeoxygenase (HO‐1 and HO‐2, respectively), which are coupled with Nrf‐2, was also significantly (*p* < .001) downregulated in ISO‐administered rats in comparison to the control rats (Figure 5b,c). These genes were significantly (Nrf‐2, *p* < .05; HO‐1 and HO‐2, *p* < .05) upregulated in response to apocynin treatment in ISO‐administered rats (Figure 5a–c).

Moreover, SOD gene expression was also declined in ISO‐administered heart compared to the control significantly (*p* < .001) (Figure 5d). The gene expression of endogenous antioxidant enzyme SOD was restored in ISO‐administered rats significantly (*p* < .001) by apocynin treatment (Figure 5d). ISO administration in rats also caused a significant (*p* < .001) decline in the mRNA level of catalase compared to the control rats. Apocynin treatment in ISO‐administered rats improved the catalase gene expression significantly (ISO vs. ISO + APO, *p* < .05) (Figure 5e). However, no significant difference was observed between control and ISO + APO groups. The gene expression of glutathione reductase was also decreased markedly (*p* < .001) in ISO‐administered rats, in relation to the control rats (Figure 5f). However, apocynin treatment did not increase the glutathione reductase gene expression significantly in ISO‐administered rats (Figure 5f).

### 3.6 Gene expression of inflammation and fibrosis‐related proteins

**FIGURE 6:**
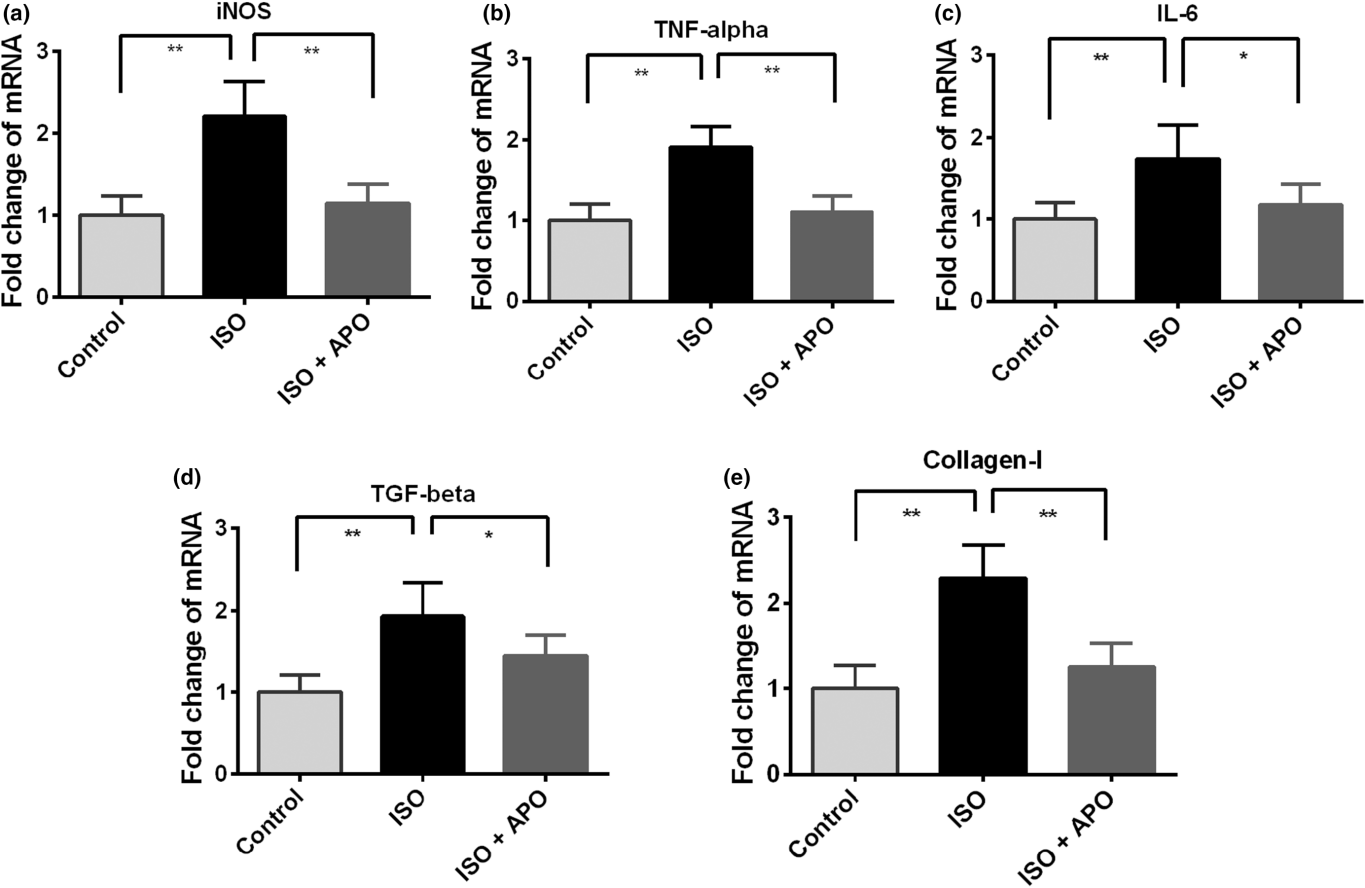
Effect of apocynin (APO) on gene expression related to inflammation in the heart of ISO‐administered rats. Data are expressed as mean ± SEM, *n* = 6. Statistical analysis was done by one‐way ANOVA followed by Tukey post hoc test. Statistical significance was considered as *p* < .05 and marked as asterisk mark. In case of *, *p* < .05 and **, *p* < .001.

The effect of apocynin on inflammation‐related genes in ISO‐administered rats was investigated through gene expression analysis by RT‐PCR. Thus, we quantified the gene expression of tumor necrosis factor‐alpha (TNF‐α), inducible nitric oxide synthase (iNOS), interleukin‐6 (IL‐6), transforming growth factor beta‐1 (TGF‐β1) and collagen‐1 to increase the validity of our results. Our investigation revealed that the gene expressions of iNOS and TNF‐α were significantly (*p* < .001) upregulated in ISO‐treated rats in comparison to control rats (Figure 6a,b). Apocynin treatment prevented the rise of iNOS and TNF‐α expression in ISO‐administered rats significantly (ISO vs. ISO + APO, *p* < .001) (Figure 6a,b). However, insignificant result was found between control and ISO + APO groups.

Further, IL‐6 and TGF‐β1 expressions were also increased considerably (*p* < .001) in ISO‐administered rats compared to the control rats (Figure 6c,d). On the other hand, significant (*p* < .05) suppressions of IL6 and TGF‐β1 expression were observed when the ISO‐administered rats were treated with apocynin (Figure 6c,d).

Moreover, the gene expression of fibrosis‐related gene collagen‐1 expression was also increased significantly (*p* < .001) in ISO‐administered rats compared to the control rats (Figure 6e). The collagen‐1 expression was subdued significantly (*p* < .001) due to apocynin treatment in ISO‐administered rats (Figure 6e).

### 3.7 Effect of apocynin on histological assessments in the heart

**FIGURE 7:**
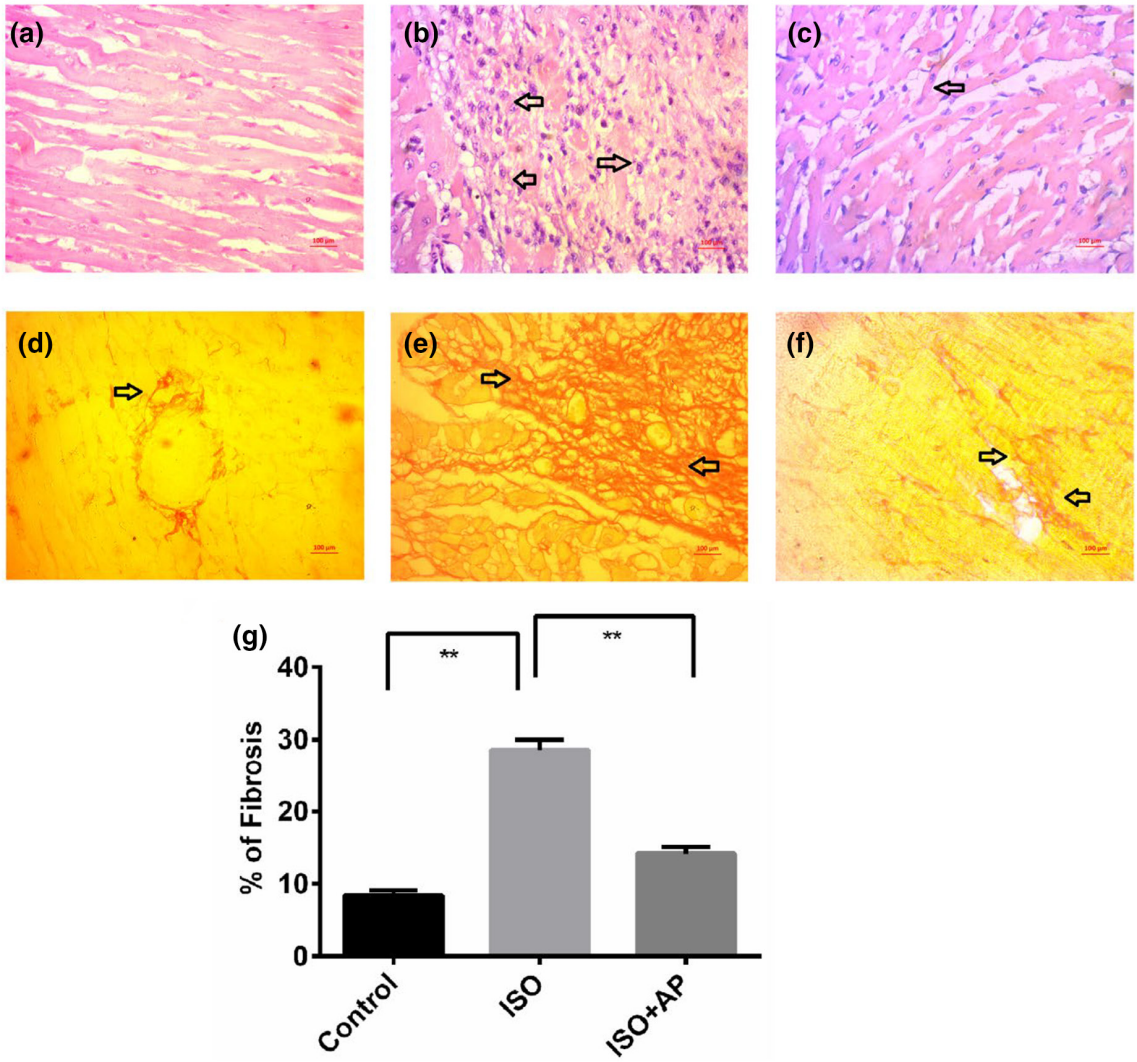
Effect of apocynin *on* inflammatory cell infiltration and fibrosis in ISO‐administered rats. (a) control group showed normal orientation of cardiomyocytes in tissue, having no infiltrating cells and necrosis. (b) ISO‐administered group showed enormous amount of infiltrating cells and necrosis (arrowhead). (c) ISO + apocynin group showed that the apocynin treatment prevented the infiltrating cells and necrosis as well as preserved the normal orientation of cardiomyocytes. The lower panel showed the Serius red staining for fibrosis. (d) Control group showed normal baseline collagen deposition in heart. (e) ISO‐administered group showed enormous amount of collagen deposition and fibrosis (arrowhead). (f) ISO + apocynin group showed that the apocynin treatment preserved the structural integrity in heart by reducing collagen deposition and fibrosis. All magnifications are of 40×. (g) Effect of apocynin (APO) on fibrosis in the heart of ISO‐administered rats. Statistical analysis was done by one‐way ANOVA followed by Tukey post hoc test. Statistical significance was considered as *p* < .05 and marked as asterisk mark. **, *p* < .001.

In this study, two histological staining techniques were performed to check the changes in tissues and necrotic damage in the heart due to ISO administration. Control rats showed normal structural formation and organization of cardiomyocytes in the left ventricle of heart visualized by H & E staining (Figure 7a). However, compared to the control rats, the infiltration of mononuclear cells in the heart tissue was also observed in ISO‐administered rats (Figure 7b). This investigation revealed that infiltration of the inflammatory cells in the left ventricle of heart was prevented by apocynin treatment in ISO‐administered rats (Figure 7c). Fibrosis and collagen deposition along with the recruitment of immune cells were found in the left ventricle of ISO‐induced rats (Figure 7d,e). Apocynin treatment prevented this collagen deposition and fibrosis in ISO‐administered rats (Figure 7f). Semiquantitative analysis also revealed that apocyanin could significantly (*p* < .001) reduce the ISO‐induced fibrosis (Figure 7g).

## 4 DISCUSSION

The present study revealed that subcutaneous administration of ISO in rats causes “infarct‐like” lesions in the heart. Similar kind of lesion is also seen in MI in humans. The characteristic changes associated with MI generally are hypertrophy of the heart, penetration of mononuclear leukocytes, deposition of collage, and fibrosis in the left ventricle of the heart (Sagor et al., 2015). We also observed that treatment with apocynin in ISO‐administered rats ameliorated oxidative stresses and inflammation. Moreover, this investigation revealed that the transcript levels of antioxidant enzymes were declined in ISO‐administered rats, which were restored by apocynin treatment.

Among the various mechanisms intricate in the pathogenesis of MI, oxidative stress‐mediated formation of MDA and lipid peroxidation is one of the most vital contributors of tissue injury in the heart (Sagor et al., 2015). In our study, apocynin decreased the lipid peroxidation levels in ISO‐administered rats. In agreement with our observations, previous findings also reported that apocynin is effective in lowering lipid peroxidation (Li et al., 2013; Liu et al., 2014; Saleem et al., 2018). The change of another oxidative stress parameter such as NO was also elevated in ISO‐administered rats, and apocynin treatment prevented the rise of NO level in ISO‐administered rats. It has been observed that the expression of iNOS increases due to the stimulation of 𝛽‐adrenergic receptors by its agonists (Balligand, 1999; Yu et al., 2019). In this study, we also observed the increased mRNA expression of iNOS in the ISO‐administered rats. This augmented gene expression of iNOS was reflected by the increased NO production. Excess NO production may create nitrosative stress reacting with other ROS such as superoxides and generates the powerful unstable structural isomer of nitrate, such as peroxynitrite (^·^ONOO^−^) (Molina‐Moya et al., 2019). Therefore, inhibition of superoxide and/or NO production may suppress peroxynitrite production, which may have beneficial outcomes in oxidative stress conditions. The molecular docking study also suggested that the interaction of apocynin with iNOS creates stable complexes, which may explain the lower level of NO in tissues of ISO‐administered rats. This result may be correlated with the previous report which showed that allicin reduced the elevated cardiac NO production as well as decreased the level of MDA and cardiac injury markers in doxorubicin‐induced mice (Abdel‐Daim et al., 2017).

There is an important association between the damage in the cardiac tissue and the release of enzymes such as alanine transaminase (ALT) and aspartate transaminase (AST). These enzymes may present in cardiac muscle and show elevated plasma activities. In our investigation treatment with apocynin resulted in a significant decline of AST, ALP, and ALT activities in the plasma. Our exploration also exhibited that heart‐specific CK‐MB activity in the plasma was also elevated significantly in ISO‐administered rats, confirming the biochemical change of acute myocardial damage. These observations agree with the previous research findings on rats showed that ISO administration increased the CK‐MB activities in plasma (Khalil et al., 2015; Ulla et al., 2017). Apocynin treatment in ISO‐administered rats decreased the plasma CK‐MB activity significantly. Moreover, infiltrating mononuclear cells containing NADPH oxidase, which is vital for the free radicals generation in a damaged heart, were also found decreased by apocynin treatment in ISO‐administered rats.

Due to the defense mechanism system of the antioxidants, a limited number of free radical may sustain in tissues. Free radical scavenging enzymes such as SOD, CAT, and GPx may reduce oxidative and nitrosative stress (Sagor et al., 2015; Ulla et al., 2017). Moreover, the diminishing of endogenous antioxidants also takes place due to ISO‐induced myocardial damage. In this study, we observed a decline of SOD and CAT activities in the heart, followed by a depletion of reduced glutathione (GSH) level in the ISO‐administered rats. This result also correlates with the findings that SOD and catalase gene expressions were declined in ISO‐administered hearts (Sagor et al., 2015; Ulla et al., 2017). Furthermore, apocynin treatment normalized the antioxidant gene expressions as well as increased the activities of SOD and catalase with the restoration of GSH level in ISO‐treated rats. A previous study also supports these findings, showing that the antioxidant enzyme levels were augmented in the heart of hyperglycemic rats by apocynin treatment (Gimenes et al., 2018). The increased mRNA expression as well as increased activities of SOD and catalase observed in this study could be attributed to the Nrf‐2‐mediated pathways. A previous study showed that Nrf‐2 mediated pathway increases the SOD and catalase enzyme‐mediated antioxidant protection in cells undergoing oxidative stress (Dreger et al., 2009).

In fact, antioxidant gene expressions were also regulated by the transcription factor Nrf‐2 (Zhu et al., 2008). Another cytoprotective enzyme, heme oxygenase, is also regulated by Nrf‐2, which catalyzes the heme molecules breakdown (Alam et al., 1999; Zhu et al., 2008). Free iron is liberated from heme and may participate in a Fenton‐like reaction for the production of more reactive hydroxyl radicle (^·^OH^−^). Nrf‐2 also modulates the expression of several other proteins, which will mask the free iron and make them unavailable for the Fenton‐like reactions (Kerins & Ooi, 2018). Earlier investigation suggests that both normoglycemic and diabetic mice may increase the infarct size and exacerbate myocardial infarction, respectively, due to a lack of HO‐1 expression (Liu et al., 2005). Conversely, HO‐1 overexpression helped to relieve left ventricular dysfunction, oxidative stress, and inflammation in streptozotocin− (STZ−) induced rodent model of diabetes (Zhao et al., 2013). The current investigation also showed that Nrf‐2 expression was downregulated in the heart which was followed by the downregulation of HO‐1 and HO‐2 expression in ISO‐administered rats. Apocynin restored the HO‐1 and HO‐2 expression through Nrf‐2‐mediated way in ISO‐administered rats.

This investigation revealed that ISO‐administered rats showed widespread myocardial structural abnormalities in the left ventricle. Free radical‐mediated cellular damage attracts inflammatory responses. ISO administration induces the entry of inflammatory leukocytes into the heart (Sagor et al., 2015). Eventually, the pro‐inflammatory genes such as iNOS, TNF‐α, and IL‐6 were increased in ISO‐administered rats which were further diminished by apocynin treatment. Moreover, the histopathological examination also showed that apocynin treatment improved the myocardial cell integrity with diminished necrosis and infiltration of inflammatory cells in the ISO‐administered rats. Researchers observed the infiltration of monocytes, macrophages, and neutrophils in the infarcted myocardium (Nahrendorf & Swirski, 2013; Puhl & Steffens, 2019). During the progression of fibrosis, the inflammatory cells contribute in the deposition of extracellular matrix (ECM) in the tissue (Frangogiannis, 2019; Lambert et al., 2008). In this study, apocynin treatment reduced the deposition of ECM in the heart of ISO‐treated rats probably by inhibiting the pro‐inflammatory cytokine expression and suppressing TGF‐β2 signaling (Hwang et al., 2016). Apocynin treatment‐mediated cardioprotection and antifibrotic activity were also supported by other studies where apocynin treatment showed ameliorative effect by preventing cardiac remodeling and fibrosis in diabetic rats (Rosa et al., 2016), angiotensin‐II‐induced hypertension in mice (Li et al., 2013), and pressure overload‐induced cardiomegaly in rats (Liu et al., 2010).

In conclusion, this investigation showed that apocynin treatment is effective and showed promising cardioprotective effects against ISO‐induced damage in the myocardium of rats. Enhancement of the antioxidant defense system, decrease in pro‐inflammatory cytokine expression, and limited inflammatory cell infiltration in the myocardium are probably the protective mechanism associated with apocynin treatment. Considering the findings of this study, more trials on apocynin for heart failure should be warranted as a translation of preclinical research to a clinical setup.

## AUTHOR CONTRIBUTIONS

**Md. Mizanur Rahman:** Formal analysis (supporting); investigation (lead); methodology (supporting); writing – original draft (supporting). **Mirza Alimullah:** Data curation (supporting); formal analysis (supporting); investigation (supporting); methodology (supporting); software (supporting); writing – original draft (supporting). **Tahmina Yasmin:** Data curation (supporting); investigation (supporting); methodology (supporting); project administration (supporting). **Nasrin Akhter:** Conceptualization (supporting); methodology (supporting); project administration (supporting); supervision (supporting); writing – original draft (supporting). **Iqbal Ahmed:** Conceptualization (supporting); formal analysis (supporting); visualization (supporting); writing – original draft (supporting); writing – review and editing (supporting). **Ferdous Khan:** Conceptualization (supporting); data curation (supporting); investigation (supporting); project administration (supporting); writing – original draft (supporting); writing – review and editing (supporting). **Mousumi Saha:** Data curation (supporting); investigation (supporting); methodology (supporting); software (supporting); writing – original draft (supporting). **Mohammad A. Halim:** Conceptualization (supporting); investigation (supporting); methodology (supporting); software (supporting); writing – original draft (supporting). **Nusrat Subhan:** Conceptualization (equal); formal analysis (supporting); investigation (supporting); methodology (supporting); project administration (supporting); supervision (supporting); writing – original draft (supporting); writing – review and editing (supporting). **Md. Areeful Haque:** Conceptualization (supporting); data curation (supporting); investigation (supporting); project administration (supporting); supervision (supporting); writing – original draft (supporting); writing – review and editing (supporting). **Md. Ashraful Alam:** Conceptualization (lead); data curation (lead); formal analysis (lead); funding acquisition (lead); project administration (lead); supervision (lead); writing – original draft (lead); writing – review and editing (lead).

## FUNDING INFORMATION

This research received a CTRG research grant from North South University Bangladesh awarded to Dr. Md. Ashraful Alam. The research was conducted in the Department of Pharmaceutical Sciences, North South University, Bangladesh.

## CONFLICT OF INTEREST STATEMENT

There is no conflict of interest.

## Data Availability

The data that support the findings of this study are available from the corresponding author upon reasonable request.
